# Data on Swiss citizens' preferences and perceptions of agricultural policy

**DOI:** 10.1016/j.dib.2024.110042

**Published:** 2024-01-11

**Authors:** Jeanine Ammann, Gabriele Mack, Judith Irek, Robert Finger, Nadja El Benni

**Affiliations:** a Agroscope, Research Group Economic Modelling and Policy Analysis, Ettenhausen, Switzerland; b ETH Zürich, Agricultural and Economic Policy Group, Zürich, Switzerland; c Agroscope, Research Division Sustainability Assessment and Agricultural Management, Ettenhausen, Switzerland

**Keywords:** Policy goal, Animal welfare, Sustainability, Farmers’ income, Consumer prices

## Abstract

We present representative survey data from 1,542 Swiss citizens. Data were collected across the three largest Swiss language regions (German, French and Italian) in autumn 2022. In the main part of the survey, we collected qualitative and quantitative data on their perception of agricultural policy goals and potential trade-offs. For this, participants were first asked to name the three most important agricultural policy goals. Next, they rated eight pre-defined agricultural policy goals for importance and how much budget they would allocate to the pursuit of each goal if they were to decide about the governmental budget or subsidies. For the goal conflicts, the eight goals were combined into 16 conflicting pairs, where participants were to decide which of the conflicting goals they preferred. Further, we collected information regarding who citizens considered as responsible for achieving agricultural policy goals. The survey was also used to collect personal information about the participating citizens including information such as gender, age, education level, place of residence or whether participant had (previous) work experience in agriculture and how they placed themselves regarding their political orientation on a left-right scale. We further collected behavioural data including diet, that is, meat consumption frequency and shopping behaviour, where we asked participants what attributes were important for them when buying food. At the end of the survey, we used existing and new scales to measure participants’ perception of farmers, meat commitment and their perception regarding animal welfare and environmental protection using the Ecological Welfare Scale. For this study, ethical approval was obtained from ETH Zurich ethical commission (application EK-2022-N-174).

Specifications Table

**Table:** 

Subject	Social Sciences, Agricultural Economics
Specific subject area	Public assessment of agricultural policy goals and trade-offs between agricultural policy goals
Data format	Raw Cleaned
Type of data	CSV file (semicolon delimited), SPSS file, survey (PDF) and codebook (PDF)
Data collection	Participants were recruited by Bilendi AG (ISO-certified panel provider) and the data were collected through an online survey (accessible from computer and phone) implemented with Tivian. Data collection took place in the German-, French- and Italian-speaking parts of Switzerland in October 2022. Quotas were used for age, gender and language region. The survey took between 15 and 20 min to complete.
Data source location	Institution: Agroscope City/Town/Region: Ettenhausen, Tänikon Country: Switzerland
Data accessibility	Repository name: ETH Zürich Research Collection Direct URL to data: https://www.research-collection.ethz.ch/handle/20.500.11850/647,439 DOI: https://doi.org/10.3929/ethz-b-000647439
Related research article	Ammann, J., Mack, G., Irek, J., Finger, R., & El Benni, N. (2023). Consumers’ meat commitment and the importance of animal welfare as agricultural policy goal Food Quality and Preference, 112(105,010). https://doi.org/10.1016/j.foodqual.2023.105010 [1]

## 1 Value of the Data

• Public acceptance is an important pillar for successful policies. With that, this data on citizens’ assessment of agricultural policy goals is of importance for both scientists and policy makers.
• The data on citizens’ perception of attributes that are important when buying food can help better understand food purchase behaviour.
• The data collected across three language regions provides a better understanding of the cultural differences within Switzerland. Multi-language surveys can be also used in other countries.
• The survey contains newly developed items measuring citizens’ perception of farmers which can be replicated and modified in other settings and further developed and validated in future studies.

## 2 Background

Agricultural policies aim to address multiple goals (e.g. food security, environmental protection, biodiversity, animal welfare, farmers’ income or consumer prices). The pursuit of specific goals often comes with conflicting objectives. For example, policies to protect the environment can cause a decline in food production or animal welfare may conflict with climate change mitigation. Thus, policy making requires setting priorities and should be aligned with societal preferences to ensure public acceptance. It is therefore crucial to know how citizens weigh the importance of different policy goals [2]. A better understanding of how preferences towards different goals are driven by sociodemographic characteristics and personal attitudes helps to better understand differences across the population and changes in preferences over time due to population development [3].

Based on this, we designed a study to collect the dataset described herein to find out which agricultural policy goals are most important to Swiss citizens. We chose Switzerland as an example of a European country with a multi-language background. With this dataset, we further aimed to better understand how personal attitudes and sociodemographic characteristics influence the preferences for different policy goals. The related research article [1] presents part of the data described herein, focussing on animal welfare.

## 3 Data Description

Data were collected through an online survey. Participant recruitment was done by a professional panel provider (Bilendi AG). We used quotas for gender (50% women), age (33% aged 18–35, 33% aged 36–54, and 33% aged 55–75), and language region (33% German, 33% French, 33% Italian). For each of the three language regions included, we aimed to recruit 500 participants. For the Italian-speaking parts, quotas had to be adapted throughout the recruiting process, as the panel provider was not able to fill the quotas as planned.

A total of 1663 participants matched the selection criteria, that is, the quotas, and completed our survey. We excluded observations from participants who took less than half the median of all participants to complete the survey, that is, less than 316 s, as we assumed that they sped through the survey. This data cleaning procedure led to the exclusion of 121 participants, resulting in a final sample size of 1542. Refer to Table 1 for an overview on the sample. Due to the survey design, participants were required to enter a response in order to proceed with the survey. As a result, there were no missing variables in the dataset. The original dataset in wide format (raw; CSV and SPSS file), the dataset after data cleaning (cleaned; CSV and SPSS file), the survey in four languages (PDF) and the codebook describing the variables (PDF) are freely available online on the ETH Zürich Research Collection: https://www.research-collection.ethz.ch/handle/20.500.11850/647439 [4].

**Table 1:** Sample description (*N* = 1542).

	German	French	Italian
	*n* = 505	*n* = 517	*n* = 520
Gender (women)	51.3	51.1	52.1
Age group			
18–35	29.7	30.2	38.7
36–54	34.5	34.6	38.1
55–75	35.8	35.2	23.3
Education			
No education, in education	0	0.4	0.6
Compulsory school	5.1	3.3	4.6
Vocational apprenticeship/vocational college/commercial (secondary) school	43.4	34.4	27.5
(Vocational) baccalaureate	10.5	12.8	20.4
Higher technical or vocational education	17.4	8.9	15.2
University of applied sciences or university of education	11.9	14.5	12.5
University	11.7	25.74	19.2
Place, where grown up			
Very rural	19.0	8.7	4.6
Rather rural	34.3	27.9	26.5
Suburban	21.8	20.5	34.0
Rather urban	13.5	25.9	22.3
Very urban	11.5	17.0	12.5
Place of residence			
Very rural	14.7	8.5	3.8
Rather rural	35	28.8	24.6
Suburban	27.7	21.5	35.6
Rather urban	12.7	26.3	24.8
Very urban	9.9	14.9	11.2
Previous and current work experience in farming			
Currently work as farmer	3.8	8.7	2.5
Used to work as farmer	10.1	11.4	11.7
Never worked as farmer	86.1	79.9	85.8

## 4 Experimental Design, Materials and Methods

We collected survey data in Switzerland in October 2022. The survey was run with the survey software Unipark and the link was sent to participants. They were able to access it online or by phone. The survey consisted of twelve distinct parts as described in the following (see also supplementary material).

Section 1: Introduction and consent

**Fig. 1:**
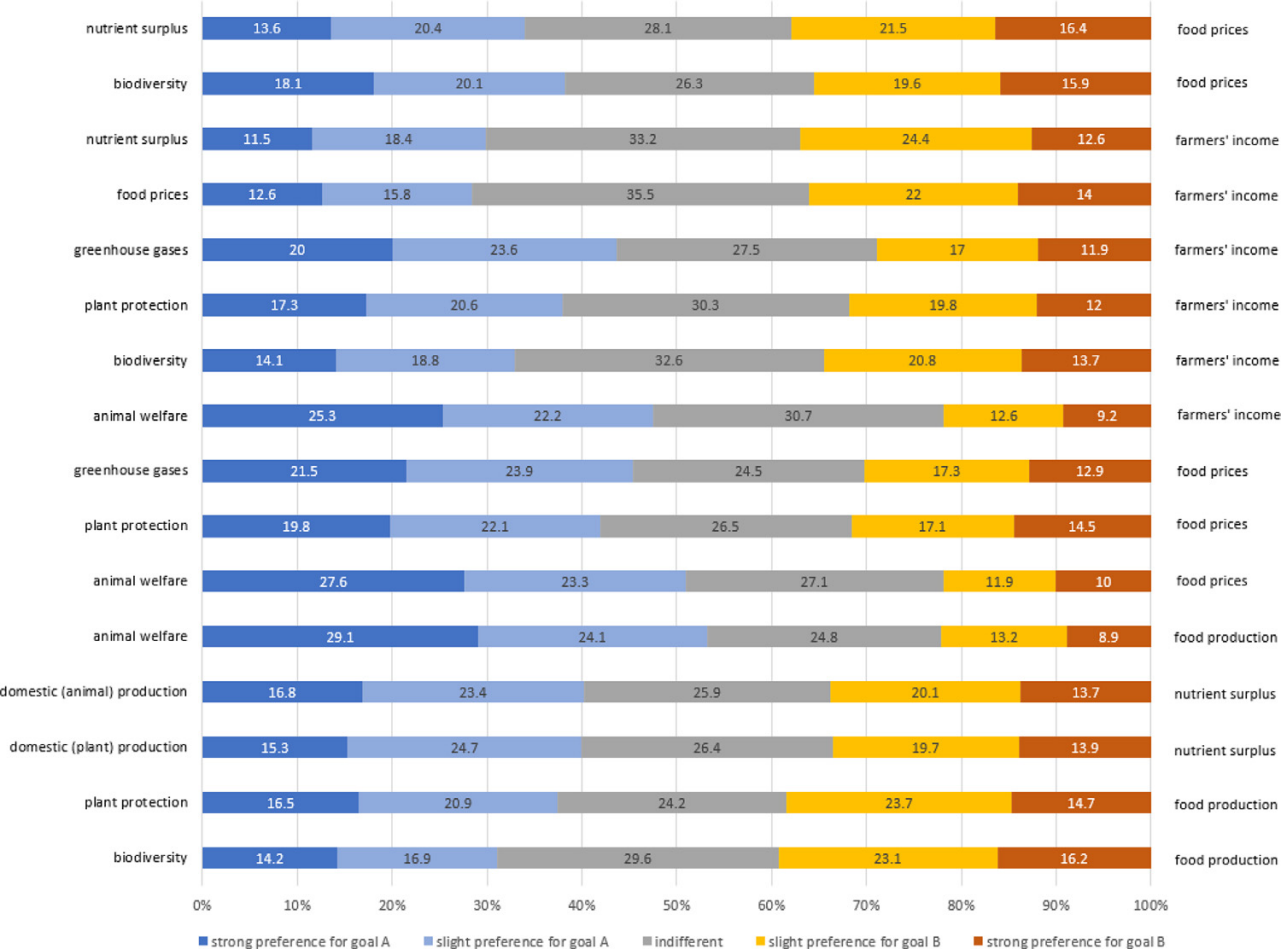
Overview on preferences for the conflicting policy goals (*N* = 1542) *Note:* Responses were given on a scale from 0 (complete preference for goal A [left]) to 100 (complete preference for the conflicting goal B [right]). For this graph, responses were grouped into five categories: 1) strong preference for goal A (0–20), 2) slight preference for goal A (21–40), 3) undecided (41–60), 4) slight preference for the conflicting goal B (61–80), and 5) strong preference for the conflicting goal B (81–100).

Section 2: Questions:

1. Personal information (see Table 1)
  a. Age
  b. Gender
  c. Education level
    i. No education, in education
    ii. Compulsory school
    iii. Vocational apprenticeship / vocational college / commercial (secondary) school
    iv. (Vocational) baccalaureate
    v. Higher technical or vocational education
    vi. University of applied sciences or university of education
    vii. University
  d. Place where grown up
  e. Current place of residence
  f. Previous and current work experience in farming
2. Diet (see Table 2)
  a. Meat consumption frequency
3. Political orientation (see Table 2)
4. Shopping behaviour (see Table 3)
  a. Importance of different attributes when shopping for food
5. Agricultural policy goals (see Table 3)
  a. Importance of selected goals
  b. Budget allocation for selected goals
6. Conflicting agricultural policy goals (see Fig. 1)
  a. Weighing of selected conflicting pairs of goals
7. Responsibility to achieve agricultural policy goals (see Table 4)
  a. Responsibility for four different players
8. Perception of farmers (see Table 5)
9. Meat commitment Scale (see Table 5)
10. Ecological Welfare Scale (see Table 5)

Section 3: Thank you and end of the survey

**Table 2:** Behavioural characteristics of the sample (*N* = 1542).

	German	French	Italian
	*n* = 505	*n* = 517	*n* = 520
Meat consumption frequency (similar to [6])			
Multiple times per day	2.4	3.5	1.3
Daily	14.9	13.5	5.6
4–6 times per week	31.3	30.8	20.8
1–3 times per week	36.2	39.3	49.8
1–3 times per month	8.9	7.2	12.9
Rarely or never	6.3	5.8	9.6
Political orientation (similar to [7])			
Left-leaning	21.8	25.1	28.5
Middle	45.5	42.7	41.2
Right-leaning	32.7	32.1	30.4

*Note.* For political orientation, participants provided their response on a scale from 0 to 100. Values from 0 to 40 were grouped as left-leaning, values between 41 and 60 as middle and values between 61 and 100 were grouped as right-leaning.

**Table 3:** Importance of various attributes when shopping for food and agricultural policy objectives (*N* = 1542).

	German		French		Italian	
	*n* = 505		*n* = 517		*n* = 520	
	M	SD	M	SD	M	SD
Important attributes when shopping for food						
Animal welfare	5.78	1.37	5.76	1.32	5.62	1.64
Environmentally friendly production	5.02	1.53	5.44	1.41	5.37	1.62
Healthy nutrition	5.72	1.25	5.98	1.19	5.27	1.53
As few additives as possible	5.45	1.46	5.75	1.42	5.42	1.67
Taste	6.26	0.92	6.31	1.00	6.08	1.34
Social standards such as fair income	5.23	1.49	5.13	1.44	4.73	1.69
Preservation and promotion of species diversity (biodiversity)	5.01	1.58	5.26	1.47	4.95	1.65
Regional origin	5.28	1.48	5.47	1.37	5.01	1.69
Price	5.64	1.26	5.63	1.30	5.67	1.50
Organic quality (label)	4.30	1.76	4.67	1.70	4.25	1.77
Importance of agricultural policy goals						
Reduce nutrient surpluses (e.g. overfertilisation)	5.63	1.39	5.75	1.24	5.45	1.52
Reduce food prices	5.36	1.50	5.63	1.40	5.68	1.54
Promote biodiversity / species diversity	5.86	1.29	5.97	1.21	5.98	1.46
Ensure adequate incomes for farmers	5.74	1.31	5.50	1.37	5.33	1.53
Reduce the use of plant protection products	4.96	1.61	5.38	1.42	5.34	1.53
Increase domestic food production	5.58	1.47	5.79	1.30	5.38	1.55
Increase animal welfare	5.69	1.14	6.19	1.03	5.84	1.36
Reduce greenhouse gas emissions	5.71	1.33	5.65	1.32	5.30	1.53
Budget for agricultural policy goals						
Reduce nutrient surpluses (e.g. overfertilisation)	5.36	1.42	5.50	1.32	5.30	1.52
Reduce food prices	5.22	1.50	5.58	1.39	5.66	1.50
Promote biodiversity / species diversity	5.75	1.31	5.86	1.23	5.88	1.48
Ensure adequate incomes for farmers	5.53	1.43	5.44	1.43	5.28	1.56
Reduce the use of plant protection products	5.02	1.69	5.37	1.43	5.40	1.46
Increase domestic food production	5.49	1.43	5.69	1.34	5.29	1.46
Increase animal welfare	5.41	1.32	6.11	1.08	5.78	1.40
Reduce greenhouse gas emissions	5.39	1.40	5.47	1.35	5.38	1.41

*Note.* Responses were given on a scale from 1 (not important at all) to 7 (very important).

**Table 4:** Responsibility of stakeholders for the achievement of agricultural policy goals (*N* = 1542).

	German		French		Italian	
	*n* = 505		*n* = 517		*n* = 520	
	M	SD	M	SD	M	SD
Farmers (e.g. through production)	5.52	1.15	5.33	1.34	4.85	1.35
Retailers (e.g. through the range of products offered by retailers)	5.61	1.15	5.57	1.33	5.08	1.32
Consumers (e.g. through consumer behaviour)	5.68	1.34	5.53	1.40	5.09	1.44
State / politics (e.g. through laws)	5.77	1.32	5.94	1.29	5.91	1.29

*Note.* Responses were given on a scale from 1 (no responsibility) to 7 (very much responsibility).

**Table 5:** Overview on participants’ attitudes towards farmer, meat and environment.

		German		French		Italian	
		*n* = 505		*n* = 517		*n* = 520	
		M	SD	M	SD	M	SD
**Perception of farmers^a^** [12,13]							
1	I am generally positive towards farmers.**^b^**	5.53	1.38	5.87	1.26	5.88	1.17
2	Farmers’ work is important and valuable for society.	6.15	1.02	6.36	1.02	6.33	0.98
3	Farmers are committed to animal welfare.**^b^**	5.04	1.41	5.38	1.35	5.02	1.42
4	Farmers have a great environmental awareness.**^b^**	4.74	1.42	4.96	1.45	4.82	1.44
5	Family farms are important and should be preserved.**^b^**	6.09	1.11	6.2	1.13	6.18	1.14
**Meat commitment^c^**[10]							
1	When I choose a menu, I almost always choose the meat option	2.98	1.97	3.03	2.04	3.27	1.97
2	The best part of many meals is the meat	4.03	2.06	3.93	2.06	3.18	1.98
3	I would never stop eating meat	4.37	2.21	4.51	2.15	3.98	2.22
4	I am a convinced meat eater	4.42	2.06	4.38	2.04	3.97	2.14
5	I don't want to eat meals without meat	3.8	1.99	3.93	2.00	3.65	2.05
6	I can't imagine stopping eating meat	4.31	2.19	4.58	2.13	3.98	2.23
7	I can't imagine replacing meat in a meal with something else	3.78	2.22	3.78	2.14	3.43	2.12
**Environmental welfare scale^d^**[11]							
It is important that the food I consume in a day ...							
1	…has been produced in a way that has not affected the balance of nature	3.47	0.70	3.42	0.73	3.43	0.73
2	…was produced in a way that does not cause pain to animals	3.38	0.70	3.32	0.76	3.39	0.72
3	…was produced in an environmentally friendly way	3.25	0.74	3.34	0.70	3.38	0.69
4	…was produced in a way that respects the rights of animals	3.22	0.69	3.28	0.74	3.28	0.68
5	…is packaged in an environmentally friendly way	3.21	0.82	3.17	0.84	3.19	0.81

*Note.*^a^: on a scale from 1 (do not agree at all) to 7 (totally agree), ^b^: Items developed in accordance with Pfeiffer, Gabriel [12], ^c^: items measured on a scale from 1 (do not agree at all) to 7 (totally agree), ^d^: items measured on a scale from 1 (not important at all) to 4 (very important).

### 4.1 Section 1: introduction and consent

In the first part of the survey, participants were briefly informed about the contents of the survey and provided their informed consent. They were informed that ethic approval was obtained from the ETH Zurich ethical commission (application EK-2022-N-174). Further, we informed them that they were free to quit the survey at any time without having to give a reason.

### 4.2 Section 2: questions

In the second part of the survey, personal information was obtained. This included participants’ age, gender, education level, place where they grew up, current place of residence. The five response categories were chosen in accordance with the terms used by the Swiss Statistical Office [5]. The breakdown of municipalities used by the Swiss Statistical Office is based on the so-called Urban/Rural Typology 2012, which separates two urban areas, that is, core cities and other urban municipalities, an intermediary settlement type with both urban and rural characteristics, and rural areas. Finally, participants were asked about their (previous) work experience in farming, to get an impression on how much they know about farming (see also Table 1).

Parts three and four of the survey assesses participants’ behaviour in terms of meat consumption frequency and political orientation. Meat consumption frequency was measured on a 6-point response scale from 1 (rarely or never) to 6 (multiple times per day), as done in previous studies [6] and described in Table 2. For participants’ political orientation, we asked them to place themselves on an interactive slider from left (0) to right (100), as done similarly by other studies including Eurobarometer [7]. The middle of the scale was marked to help participants orient themselves. However, no start position was given for the curser in order not to influence participants. The curser only appeared after they clicked on the interactive slider.

To investigate participants’ food shopping behaviour, we asked participants in part five of the survey to rate a list of ten attributes for importance when buying food, which was chosen in accordance with the Swiss Biobarometer, which is a regular survey on organic consumption in Switzerland [8]. Participants provided their responses on a 7-point response scale from 1 (not important at all) to 7 (very important). The mean responses are shown in Table 3.

Next, in parts six, seven and eight of the survey, we collected data related to the perception of agricultural policy. Specifically, we measured participants’ perception of agricultural policy goals qualitatively and quantitatively. In a first step, we asked participants to name the three most important agricultural policy goals for Switzerland. They were free to write down whatever came to their mind in three text fields provided. Next, we provided them with a list of eight agricultural policy goals. Those were chosen in accordance with accordance with Article 104 of the Swiss Constitution [9], which defines the goals of Swiss agriculture agricultural production.

Participants were asked to rate each of the eight selected policy goals for importance on a 7-point response scale from 1 (not important at all) to 7 (very important, Table 3). After that, we instructed participants to imagine that they were responsible for the agricultural policy budget in Switzerland. Based on this assumption, we asked them to indicate how important each of the eight policy goals was for the distribution the available budget or subsidies, again on a 7-point response scale from 1 (not important at all) to 7 (very important, Table 3).

Based on the eight agricultural policy goals, we then defined sixteen possible goal conflicts. For each conflict, participants indicated on an interactive slider scale from 0 (strong preference for the first goal) to 100 (strong preference for the second goal), which agricultural policy goal they preferred (Fig. 1). Importantly, no starting position for the curser on the interactive slider scale was given. With that, we made sure that participants were not influenced by the starting position of the curser. It only appeared after they clicked on the slider scale. Further, the presentation order of the sixteen conflicts was randomized to control for order effects.

In part nine of the survey, we asked participants for four stakeholder groups how much responsibility they had to make sure the agricultural policy goals were achieved (Table 4). Responses were given on a 7-point scale from 1 (no responsibility) to 7 (high responsibility).

Finally, in parts ten, eleven and twelve, we measured participants’ attitudes towards farmers, meat and ecological welfare. Table 5 summarizes the three constructs with the corresponding items. For the perception of farmers, participants rated five statements on a scale from 1 (do not agree at all) to 7 (totally agree) for their agreement. To investigate how committed participants were to eating meat, we used the seven statements as developed by Piazza, Ruby [10], which participants rated on a scale from 1 (do not agree at all) to 7 (totally agree). Finally, participants filled in the Ecological Welfare Scale by Lindeman and Vaananen [11], which consists of five items and covers both animal welfare and environmental protection. These statements were assessed on a scale from 1 (not important at all) to 4 (very important).

### 4.3 Section 3: thank you and end of the survey

In the final part of the survey, participants were given the possibility to write down any comments if they wished to do so. After that, we thanked participants for their participation and they were instructed to close the survey.

### 4.4 Experimental design and response variables

The key part of this study are parts seven and eight of the survey, where participants rated eight policy goals and assessed sixteen possible goal conflicts. We defined eight agricultural policy goals and combined them into sixteen pairs, each describing a goal conflict. Each of the eight goals was rated for importance on a scale from 1 (not important at all) to 7 (very important). Each of the sixteen goal conflicts was rated for preference on a scale from 0 (strong preference for the first goal) to 100 (strong preference for the second goal). This data quality was chosen to make sure that the response variables were suitable for regression analyses.

## Limitations

One limitation of this data is that for the assessment of participants’ preference for certain agricultural policy goals, the complex relationships that agricultural policy entails had to be broken down into more simple contexts. Further, participants evaluated pairs conflicting agricultural policy goals. In reality, the relationships are more complex and the pursuit of one goal will most certainly affect more than one conflicting goal. Another limitation to mention is that we were not able to fill the quotas as planned for the Italian language region regarding age (see Table 1). That is, we were not able to find sufficient participants for the oldest age group. This has to be considered if the language groups are to be compared.

## Ethics Statement

All participants involved in the study provided their written, informed consent to participate. Participation was voluntary and could be withdrawn at any time. Participants remained anonymous and their responses were dealt with in confidence. Ethical approval was obtained from the ethical commission at ETH Zurich (application EK-2022-N-174).

## CRediT authorship contribution statement

**Jeanine Ammann:** Conceptualization, Investigation, Data curation, Writing – original draft, Project administration. **Gabriele Mack:** Conceptualization, Methodology, Writing – review & editing. **Judith Irek:** Conceptualization, Methodology, Writing – review & editing. **Robert Finger:** Conceptualization, Methodology, Writing – review & editing. **Nadja El Benni:** Conceptualization, Methodology, Writing – review & editing, Resources.

## Data Availability

Dataset: Swiss citizens preferences and perceptions of agricultural policy (Original data) (ETH Research Collection)

## Appendix Supplementary materials

Image, application 1
