# Supplementary material for: Data on Swiss citizens' preferences and perceptions of agricultural policy
Source: Data Brief. 2024 Jan 11;52:110042. doi: 10.1016/j.dib.2024.110042 (PMC10831490; doi:10.1016/j.dib.2024.110042)
Supplement: Supplementary file 1 [file mmc1.docx]

**Supplementary material**

Table 1: Survey and coding in German (original) and its English translation

|  | **German (original)** | **English translation** | |
| --- | --- | --- | --- |
| **Introduction** | | |  |
|  | Warum werde ich gebeten, an dieser Forschungsstudie teilzunehmen?  Wir laden Sie ein, an einer Studie teilzunehmen, in der wir mehr über Ihre Meinung zur Schweizer Agrarpolitik herausfinden möchten. Sie müssen mindestens 18 Jahre alt sein, um an der Umfrage teilnehmen zu können.  Was ist der Zweck dieser Studie?  Die Umfrage soll neue wissenschaftliche Erkenntnisse über die Agrarpolitik in der Schweiz hervorbringen. Diese Studie ist eine Zusammenarbeit zwischen Agroscope, dem Kompetenzzentrum des Bundes für die landwirtschaftliche Forschung und der ETH Zürich.  Wie viel Zeit muss ich investieren?  Die Teilnahme an der Umfrage dauert ca. 15-20 Minuten.  Was ist der genaue Inhalt der Umfrage?  Sie werden gebeten, verschiedene agrarpolitische Ziele zu gewichten. Sie beantworten zudem einige soziodemografische Fragen.  Was passiert, wenn ich nicht teilnehmen möchte?  Ihre Teilnahme an dieser Studie ist freiwillig. Sie können sich entscheiden, nicht an dieser Studie teilzunehmen. Zudem können Sie die Befragung jederzeit verlassen.  Könnte mir die Teilnahme an dieser Studie irgendwie schaden?  Diese Umfrage enthält keine Fragen, die Ihnen ein Risiko bringen oder Ihnen Unbehagen bereiten könnten. Sie können jedoch jede Frage, die Sie nicht beantworten möchten, überspringen, oder die Umfrage an jeder Stelle abbrechen.  Was geschieht mit den für die Forschung gesammelten Informationen?  Es werden keine direkten persönlichen Merkmale erfasst. Ihre Angaben werden im Rahmen der gesetzlichen Bestimmungen immer vertraulich behandelt. Die Ergebnisse und die Daten der Forschungsstudie können veröffentlicht werden, Ihre Identität bleibt jedoch immer anonym. Ihre Daten werden Forschenden von Agroscope und der ETH Zürich zugänglich sein.  Mit wem kann ich sprechen?  Fragen zu dieser Studie können gerne an die Studienleitung (Dr. Jeanine Ammann, jeanine.ammann@agroscope.admin.ch) gerichtet werden. Sie können sich auch an das Sekretariat Ethikkommission der ETH Zürich wenden, telefonisch unter +41 44 63 28572 oder per E-Mail unter ethics@sl.ethz.ch. | Why am I being asked to take part in this research study?  We are inviting you to take part in a study to find out more about your opinion on Swiss agricultural policy. You must be at least 18 years old to take part in the survey.  What is the purpose of this study?  The survey aims to generate new scientific knowledge about agricultural policy in Switzerland. This study is a collaboration between Agroscope, the federal government's centre of excellence for agricultural research, and ETH Zurich.  How much time do I have to invest?  Participation in the survey takes about 15-20 minutes.  What is the exact content of the survey?  You will be asked to give weight to various agricultural policy objectives. You will also answer some socio-demographic questions.  What happens if I do not want to participate?  Your participation in this survey is voluntary. You can decide not to take part in this study. Furthermore, you can leave the survey at any time.  Could participating in this study harm me in any way?  This survey does not contain any questions that could put you at risk or make you feel uncomfortable. However, you can skip any question you do not want to answer or stop the survey at any point.  What happens to the information collected for the research?  No direct personal characteristics are collected. Your information will always be kept confidential within the limits of the law. The results and data of the research study may be published, but your identity will always remain anonymous. Your data will be accessible to researchers from Agroscope and ETH Zurich.  Who can I talk to?  Questions about this study can be addressed to the study director (Dr Jeanine Ammann, jeanine.ammann@agroscope.admin.ch). You can also contact the ETH Zurich Ethics Committee Secretariat by phone at +41 44 63 28572 or by e-mail at ethics@sl.ethz.ch. | |
| **Consent** | | |  |
|  | Wenn Sie eine Kopie dieser Zustimmung für Ihre Unterlagen benötigen, können Sie sie ausdrucken.  Wenn Sie teilnehmen möchten, klicken Sie bitte auf die Schaltfläche "Ich stimme zu" und Sie werden zur Umfrage weitergeleitet.  Wenn Sie nicht an dieser Studie teilnehmen möchten, wählen Sie bitte "Ich stimme nicht zu" oder wählen Sie X in der Ecke Ihres Browsers.  Mit Ihrer Zustimmung, bestätigen Sie, dass Sie die obige Erklärung gelesen haben und die Möglichkeit hatten, Fragen zu stellen und Bedenken zu äussern. Sie bestätigen, dass Sie den Zweck der Studie sowie die damit einhergehenden potenziellen Risiken verstehen. Sie bestätigen, dass Ihre Teilnahme freiwillig ist und dass Sie mit Ihrer Zustimmung auf keine Rechte verzichten. Sie bestätigen, dass Sie mindestens 18 Jahre alt sind. Ihre Teilnahme an der Studie können Sie jederzeit beenden.  Wenn Sie mit der obigen Erklärung einverstanden sind, klicken Sie auf "Ich stimme zu, beginne die Studie" und auf «weiter», um fortzufahren. | If you need a copy of this consent for your records, you can print it out.  If you wish to participate, please click on the "I agree" button and you will be redirected to the survey.  If you do not wish to participate in this study, please select "I do not agree" or select X in the corner of your browser.  By agreeing, you acknowledge that you have read the above statement and have had the opportunity to ask questions and express concerns. You acknowledge that you understand the purpose of the study and the potential risks involved. You confirm that your participation is voluntary and that you do not waive any rights by giving your consent. You confirm that you are at least 18 years of age. You can end your participation in the study at any time.  If you agree with the above statement, click on "I agree, start the study" and on "continue" to proceed. | |
| [v_2] | Ich stimme zu und beginne mit der Studie | I agree and start the study | |
| [v_432] | Ich stimme nicht zu und möchte nicht an der Studie teilnehmen. | I do not agree and do not wish to participate in the study. | |
| **Personal information** | | |  |
|  | Wir bitten Sie nun einige Angaben zu Ihrer Person zu machen. | We now ask you to provide some information about yourself. | |
| [v_141] | Bitte geben Sie uns Ihr Geschlecht an.  -Mann [1]  -Frau [2]  -Anderes [3]  -Möchte nicht antworten [4] | Please tell us your gender.  -Male [1]  -Woman [2]  -Other [3]  -Do not wish to answer [4] | |
| [v_143] | Bitte geben Sie uns Ihr Geburtsjahr im Format JJJJ an. | Please give us your year of birth in the format YYYY. | |
| [v_144] | Kreuzen Sie bitte Ihre höchste, abgeschlossene Ausbildung an.  -Kein Abschluss / in Ausbildung [1]  -Obligatorische Schule [2]  -Berufslehre / Berufsfachschule / Handels(mittel)schule [3]  -Maturität / Berufsmaturität [4]  -Höhere Fach- oder Berufsausbildung [5]  -Fachhochschule oder pädagogische Hochschule [6]  -Universität / ETH [7] | Please tick your highest completed education.  -No degree / in education [1]  -Compulsory school [2]  -Vocational apprenticeship / Vocational school / Commercial (secondary) school [3]  -Matura / vocational baccalaureate [4]  -Higher technical or vocational training [5]  -University of applied sciences or university of teacher education [6]  -University / ETH [7] | |
| [v_433] | Wie würden Sie die Gegend beschreiben, in der Sie aufgewachsen sind?  -Sehr ländlich [1]  -Eher ländlich [2]  -Vorstädtisch [3]  -Eher städtisch [4]  -Sehr städtisch [5] | How would you describe the area where you grew up?  -Very rural [1]  -Very rural [2]  -Suburban [3]  -Probably urban [4]  -Very urban [5] | |
| [v_145] | Was trifft am ehesten auf Ihre aktuelle Wohngegend zu?  -Sehr ländlich [1]  -Eher ländlich [2]  -Vorstädtisch [3]  -Eher städtisch [4]  -Sehr städtisch [5] | Which is most likely to apply to your current neighbourhood?  -Very rural [1]  -Very rural [2]  -Suburban [3]  -Probably urban [4]  -Very urban [5] | |
| **Previous and current work experience in farming** | | | |
| [v_147] | Sind Sie / waren Sie Landwirt/in?  - Ja, derzeit als Landwirt/in aktiv.[1]  - Nein, heute nicht mehr, aber ich war zu einem früheren Zeitpunkt aktiv.[2]  - Nein, war nie als Landwirt/in tätig.[3] | Are you/were you a farmer?  - Yes, currently active as a farmer.[1]  - No, not today, but I was active at an earlier stage.[2]  - No, never worked as a farmer. [3] | |
| **Diet** | |  | |
| [v_148] | Wie oft essen Sie Fleisch?  -Mehrmals täglich [1]  -Täglich [2]  -4-6 mal pro Woche [3]  -1-3 mal pro Woche [4]  -1-3 mal pro Monat [5]  -Selten oder gar nie [6] | How often do you eat meat?  -Multiple times a day [1]  -Daily [2]  -4-6 times per week [3]  -1-3 times per week [4]  -1-3 times per month [5]  -Rarely or never[6] | |
| [v_197] | Wo ordnen Sie sich auf einer politischen links-rechts Skala ein?  Bitte klicken Sie in den Balken, um Ihre Antwort abzugeben.  Ganz links [0] – Mitte [50] – ganz rechts [100]  [scale from 0-100] | Where do you rank yourself on a political left-right scale?  Please click in the bar to give your answer.  Far left [0] – centre [50] - far right [100]  [scale from 0-100] | |
| **Shopping behaviour** | | |  |
| Nun folgen ein paar Fragen zu Ihrem Einkaufsverhalten. | | | Now follow a few questions about your shopping behaviour. |
|  | Wie wichtig sind Ihnen nachfolgende Aspekte beim Lebensmitteleinkauf?  Gar nicht wichtig [1] – neutral [4] – sehr wichtig [7] | How important are the following aspects to you when buying food?  Not at all important [1] - Neutral [4] - Very important [7] | |
| [v_163] | - Artgerechte Tierhaltung | - Animal welfare | |
| [v_166] | - Umwelt- / klimaschonende Produktion | - Environmentally / climate friendly production | |
| [v_167] | - Gesunde Ernährung | - Healthy nutrition | |
| [v_168] | - Möglichst wenige Zusatzstoffe | - As few additives as possible | |
| [v_169] | - Geschmack | - Taste | |
| [v_246] | - Sozialstandards wie z.B. faire Einkommen | - Social standards such as fair incomes | |
| [v_247] | - Erhaltung und Förderung der Artenvielfalt (Biodiversität) | - Preservation and promotion of species diversity (biodiversity) | |
| [v_248] | - Regionale Herkunft | - Regional origin | |
| [v_250] | - Preis | - Price | |
| [v_370] | - Bioqualität (Bio-Label) | - Organic quality (organic label) | |
| **Agricultural policy** | | |  |
|  | Die Agrarpolitik bzw. die landwirtschaftliche Produktion in der Schweiz verfolgt verschiedene Ziele. Bitte nennen Sie nachfolgend drei agrarpolitische Ziele, die Ihrer Meinung nach am wichtigsten sind. | Agricultural policy or agricultural production in Switzerland pursues various objectives. Please name three agricultural policy goals below that you consider to be the most important. | |
| [v_382] | Ziel 1: ___________ | Goal 1: ___________ | |
| [v_383] | Ziel 2: ___________ | Goal 2: ___________ | |
| [v_384] | Ziel 3: ___________ | Goal 3: ___________ | |
|  | Bitte geben Sie für die nachfolgenden Aspekte an, wie wichtig diese Ihrer Meinung nach für die Landwirtschaft in der Schweiz sein sollten.  Gar nicht wichtig [1] – neutral [4] – sehr wichtig [7] | Please indicate for the following aspects how important you think they should be for agriculture in Switzerland.  Not at all important [1] - Neutral [4] - Very important [7] | |
| [v_371] | - Artenvielfalt / Biodiversität fördern | - Promote biodiversity | |
| [v_372] | - Treibhausgasemissionen reduzieren | - Reduce greenhouse gas emissions | |
| [v_373] | - Tierwohl erhöhen | - Increase animal welfare | |
| [v_374] | - Nahrungsmittelproduktion im Inland erhöhen | - Increase domestic food production | |
| [v_375] | Lebensmittelpreise senken | - Reduce food prices | |
| [v_376] | -Pflanzenschutzmitteleinsatz reduzieren | - Reduce the use of plant protection products | |
| [v_377] | Angemessene Einkommen für LandwirtInnen sicherstellen | - Ensure adequate incomes for farmers | |
| [v_380] | -Nährstoffüberschüsse (z.B. Überdüngung) reduzieren | -Reduce nutrient surpluses (e.g. overfertilisation) | |
| **Budget** | | |  |
|  | Stellen Sie sich vor, Sie könnten über das Agrarbudget in der Schweiz verfügen. Bitte geben Sie für die nachfolgenden Aspekte an, wie wichtig diese bei der Verteilung des Agrarbudgets (bzw. der Subventionen) sein sollten.  Gar nicht wichtig [1] – neutral [4] – sehr wichtig [7] | Imagine that you could dispose of the agricultural budget in Switzerland. Please indicate for the following aspects how important they should be in the distribution of the agricultural budget (or subsidies).  Not at all important [1] - Neutral [4] - Very important [7] | |
| [v_435] | - Artenvielfalt / Biodiversität fördern | - Promote biodiversity | |
| [v_436] | - Treibhausgasemissionen reduzieren | - Reduce greenhouse gas emissions | |
| [v_437] | - Tierwohl erhöhen | - Increase animal welfare | |
| [v_438] | - Nahrungsmittelproduktion im Inland erhöhen | - Increase domestic food production | |
| [v_439] | Lebensmittelpreise senken | - Reduce food prices | |
| [v_440] | -Pflanzenschutzmitteleinsatz reduzieren | - Reduce the use of plant protection products | |
| [v_441] | Angemessene Einkommen für LandwirtInnen sicherstellen | - Ensure adequate incomes for farmers | |
| [v_442] | -Nährstoffüberschüsse (z.B. Überdüngung) reduzieren | -Reduce nutrient surpluses (e.g. overfertilisation) | |
| **Conflicting agricultural policy goals** | | |  |
|  | Viele agrarpolitische Ziele stehen in direktem Konflikt mit anderen Zielen. In diesem Teil des Fragebogens sollen Sie jeweils zwei vorgegebene Ziele, die in direktem Konflikt zueinander stehen, gegeneinander gewichten.  Bitte beachten Sie, dass wir uns hier auf die Situation in der Schweiz beschränken. Importe und Exporte werden nicht berücksichtigt. Ausserdem geht es um generelle Zusammenhänge, die in Einzelfällen nicht zwingend zutreffen müssen.  Bitte klicken Sie in den Balken, um Ihre Antwort abzugeben | Many agricultural policy goals are in direct conflict with other goals. In this part of the questionnaire, you are asked to weight two given goals that are in direct conflict with each other.  Please note that we are limiting ourselves here to the situation in Switzerland. Imports and exports are not taken into account. Furthermore, we are dealing with general correlations that do not necessarily apply in individual cases.  Please click in the bar to submit your answer. | |
| [v_396] | Mehr Flächen zur Förderung der Biodiversität (z.B. Hecken, Blühstreifen, Hochstammobstbäume, extensive Wiesen und Weiden) bedeuten weniger Flächen zur inländischen Nahrungsmittelproduktion, weil die verfügbaren Flächen insgesamt begrenzt sind.  -Mehr Fläche für Biodiversität /  mehr Fläche für inländische Nahrungsmittelproduktion | More land for the promotion of biodiversity (e.g. hedges, flowering strips, standard fruit trees, extensive meadows and pastures) means less land for domestic food production, because the available land is limited overall.  - More land for biodiversity / more land for domestic food production | |
| [v_397] | Mehr Pflanzenschutzmittel bedeuten höhere und stabilere Produktionsmengen im Pflanzenbau im Inland, weil Ernteausfälle durch Schädlingsbefall verhindert werden können.  -weniger Pflanzenschutzmitteleinsatz / mehr inländische Nahrungsmittelproduktion | More crop protection products mean higher and more stable domestic crop production volumes because crop failures due to pest infestation can be prevented.  - Less use of crop protection products / more domestic food production | |
| [v_398] | Mehr / intensivere pflanzliche Produktion bedeutet mehr Nährstoffüberschüsse, weil durch höhere Düngergaben höhere Erntemengen erzielt werden.  -mehr inländische Nahrungsmittelproduktion /  weniger Nährstoffüberschüsse | More / more intensive crop production means more nutrient surpluses because higher fertiliser applications result in higher crop yields.  - More domestic food production /  fewer nutrient surpluses | |
| [v_399] | Mehr tierische Produktion bedeutet mehr Nährstoffüberschüsse, weil mehr Gülle und Mist anfallen.  -mehr inländische Nahrungsmittelproduktion /  weniger Nährstoffüberschüsse | More animal production means more nutrient surpluses because more manure and slurry are produced.  - More domestic food production /  fewer nutrient surpluses | |
| [v_400] | Mehr Tierwohl (z.B. Freilandhaltung) bedeutet weniger Inlandproduktion, weil der Platzbedarf pro Tier höher ist.  -mehr Tierwohl /  mehr inländische Produktion | More animal welfare (e.g. free-range) means less domestic production because the space required per animal is higher.  - More animal welfare /  more domestic production | |
| [v_401] | Mehr Tierwohl bedeutet höhere Lebensmittelpreise, weil artgerechte Haltung mit Mehrkosten verbunden ist.  -mehr Tierwohl /  tiefere Lebensmittelpreise | More animal welfare means higher food prices, because species-appropriate husbandry is associated with additional costs.  - More animal welfare /  lower food prices | |
| [v_402] | Weniger Pflanzenschutzmittel-Einsatz verringert die Nahrungsmittelproduktion und führt damit zu höheren Lebensmittelpreisen für KonsumentInnen.  -weniger Pflanzenschutzmittel-Einsatz/  tiefere Lebensmittelpreise | Less use of plant protection products reduces food production and thus leads to higher food prices for consumers.  - Less use of plant protection products/  lower food prices | |
| [v_403] | Die Vermeidung von Treibhausgas-Emissionen erhöht die Produktionskosten und führt damit zu höheren Lebensmittelpreisen für KonsumentInnen.  -weniger Treibhausgas-Emissionen / tiefere Lebensmittelpreise | Avoiding greenhouse gas emissions increases production costs and thus leads to higher food prices for consumers.  - Less greenhouse gas emissions / lower food prices | |
| [v_404] | Mehr Tierwohl bedeutet höhere Produktionskosten und damit geringeres Einkommen der LandwirtInnen.  -Mehr Tierwohl /  höhere landwirtschaftliche Einkommen | More animal welfare means higher production costs and thus lower income for farmers.  - More animal welfare / higher farm incomes | |
| [v_405] | Mehr Biodiversität bedeutet höhere Produktionskosten und damit geringeres Einkommen der LandwirtInnen.  -Mehr Biodiversität /  höhere landwirtschaftliche Einkommen | More biodiversity means higher production costs and thus lower income for farmers.  - More biodiversity / higher agricultural income | |
| [v_406] | Weniger Pflanzenschutzmittel-Einsatz bedeutet höhere Produktionskosten, was zu tieferen Einkommen der LandwirtInnen führt.  -weniger Pflanzenschutzmitteleinsatz/ höhere landwirtschaftliche Einkommen | Less use of plant protection products means higher production costs, which leads to lower incomes for farmers.  - Less plant protection product use / higher farm incomes | |
| [v_407] | Die Vermeidung von Treibhausgas-Emissionen erhöht die Produktionskosten und führt damit zu geringerem Einkommen der LandwirtInnen.  -weniger Treibhausgasemissionen / höhere landwirtschaftliche Einkommen | Avoiding greenhouse gas emissions increases production costs and thus leads to lower income for farmers.  - Less greenhouse gas emissions / higher farm incomes | |
| [v_408] | Niedrigere Lebensmittelpreise für die KonsumentInnen können zu weniger hohen Einkommen bei den LandwirtInnen führen.  -tiefere Lebensmittelpreise/  höhere landwirtschaftliche Einkommen | Lower food prices for consumers can lead to less income for farmers.  - Lower food prices/  higher farm incomes | |
| [v_409] | Weniger Nährstoffüberschüsse erfordern reduzierte Düngergaben und können somit zu geringeren Erntemengen und damit zu kleineren Einkommen der LandwirtInnen führen.  -weniger Nährstoffüberschüsse/ höhere landwirtschaftliche Einkommen | Fewer nutrient surpluses require reduced fertiliser applications and can thus lead to lower crop yields and thus lower incomes for farmers.  - Less nutrient surpluses/ higher farm incomes | |
| [v_410] | Mehr Biodiversität (z.B. Hecken, Blühstreifen etc.) bedeutet höhere Lebensmittelpreise, weil Förderung der Artenvielfalt mit Mehrkosten verbunden ist.  -mehr Biodiversität / tiefere Lebensmittelpreise | More biodiversity (e.g. hedges, flower strips, etc.) means higher food prices, because promoting biodiversity involves additional costs.  - More biodiversity/ lower food prices | |
| [v_411] | Weniger Nährstoffüberschüsse erfordern reduzierte Düngergaben und können somit zu geringeren Erntemengen und damit zu höheren Preisen für Konsument/innen führen.  -weniger Nährstoffüberschüsse /  tiefere Lebensmittelpreise | Fewer nutrient surpluses require reduced fertiliser applications and can thus lead to lower harvests and thus to higher prices for consumers.  - Less nutrient surpluses/  lower food prices | |
| **Responsibility** | | |  |
|  | Wenn es um das Erreichen agrarpolitischer Ziele geht, bei wem sehen Sie da die grösste Verantwortung? Wer muss Ihrer Meinung nach dafür sorgen, dass die Ziele erreicht werden?  Gar keine Verantwortung [1] – neutral [4] – sehr viel Verantwortung [7] | When it comes to achieving agricultural policy goals, who do you see as having the greatest responsibility? Who do you think must ensure that the goals are achieved?  No responsibility at all [1] - Neutral [4] - Very much responsibility [7] | |
| [v_423] | Produktion / Landwirtschaft (z.B. über die Produktion) | - Production / agriculture (e.g. through production) | |
| [v_434] | -Handel (z.B. über das Angebot beim Detailhändler) | - Retailers (e.g. through the range of products offered by retailers) | |
| [v_424] | -Konsument/innen (z.B. über Konsumverhalten) | - Consumers (e.g. through consumer behaviour) | |
| [v_421] | -Staat / Politik (z.B. über Gesetze, Subventionen) | - State / politics (e.g. through laws, subsidies) | |
| **Perception of farmers** | | |  |
|  | Bitte geben Sie für die nachfolgenden Aussagen an, wie sehr Sie diesen jeweils zustimmen.  Stimme gar nicht zu [1] – weder noch [4] – stimme voll und ganz zu [7] | For the following statements, please indicate how much you agree with each of them.  Disagree [1] - Neither [4] - Strongly agree [7] | |
| [v_214] | Ich bin LandwirtInnen gegenüber generell positiv eingestellt | I have a generally positive attitude towards farmers | |
| [v_215] | Die Arbeit der LandwirtInnen ist wichtig und wertvoll für die Gesellschaft. | The work of farmers is important and valuable for society. | |
| [v_216] | LandwirtInnen setzen sich für das Tierwohl ein. | Farmers are committed to animal welfare. | |
| [v_337] | LandwirtInnen haben ein grosses Umweltbewusstsein. | Farmers have a high level of environmental awareness. | |
| [v_338] | Bäuerliche Familienbetriebe sind wichtig und sollten erhalten bleiben. | Family farms are important and should be preserved. | |
| **Meat consumption** | | |  |
|  | Bitte geben Sie für die nachfolgenden Aussagen an, wie sehr Sie diesen jeweils zustimmen.  Stimme gar nicht zu [1] – stimme voll und ganz zu [7] | For the following statements, please indicate how much you agree with each of them.  Strongly disagree [1] - Strongly agree [7] | |
| [v_349] | -Ich möchte keine Gerichte ohne Fleisch essen | - I don't want to eat meals without meat | |
| [v_350] | -Wenn ich ein Menü auswähle, wähle ich praktisch immer die Fleischvariante | - When I choose a menu, I almost always choose the meat option | |
| [v_351] | -Ich kann mir nicht vorstellen, mit dem Fleischessen aufzuhören | - I can't imagine stopping eating meat | |
| [v_352] | -Ich bin überzeugte/r Fleischesser/in | - I am a convinced meat eater | |
| [v_353] | -Der beste Teil vieler Mahlzeiten ist das Fleisch | - The best part of many meals is the meat | |
| [v_354] | -Ich würde niemals aufhören, Fleisch zu esse | - I would never stop eating meat | |
| [v_355] | -Ich kann mir nicht vorstellen, Fleisch in einer Mahlzeit durch etwas anderes zu ersetzen | - I can't imagine replacing meat in a meal with something else | |
| **Food** | | |  |
|  | Es ist wichtig, dass die Lebensmittel, die ich an einem Tag konsumiere, …  Gar nicht wichtig [1] – sehr wichtig [4] | It is important that the food I consume in a day is ...  Not at all important [1] - Very important [4] | |
| [v_356] | in einer Art und Weise produziert wurden, dass Tiere keine Schmerzen erfahren haben | produced in a way that does not cause pain to animals | |
| [v_357] | in einer Art und Weise produziert wurden, dass die Rechte der Tiere respektiert wurden | are produced in a way that respects the rights of animals | |
| [v_358] | umweltfreundlich produziert wurden | were produced in an environmentally friendly way | |
| [v_359] | in einer Art und Weise hergestellt wurden, dass das Gleichgewicht der Natur nicht beeinträchtigt wurde | have been produced in a way that has not affected the balance of nature | |
| [v_360] | umweltfreundlich verpackt sind | are packaged in an environmentally friendly way | |
| **End** | | |  |
|  | Wir sind schon fast am Ende dieser Befragung. Sollten Sie noch weitere Bemerkungen haben, können Sie diese hier notieren.  Vielen Dank!  Somit sind wir jetzt am Ende dieser Befragung angelangt.  Wir danken Ihnen ganz herzlich für Ihre wertvolle Teilnahme an dieser Studie.  Sie können die Umfrage jetzt schliessen. | We are almost at the end of this survey. If you have any further comments, you can write them down here.  Thank you very much!  We have now reached the end of this survey.  We thank you very much for your valuable participation in this survey.  You can now close the survey | |
